# *In Vitro* Activity of Neomycin, Streptomycin, Paromomycin and Apramycin against Carbapenem-Resistant *Enterobacteriaceae* Clinical Strains

**DOI:** 10.3389/fmicb.2017.02275

**Published:** 2017-11-17

**Authors:** Ya Hu, Lu Liu, Xiaoxia Zhang, Yu Feng, Zhiyong Zong

**Affiliations:** ^1^Center of Infectious Diseases, West China Hospital, Sichuan University, Chengdu, China; ^2^Division of Infectious Diseases, State Key Laboratory of Biotherapy, Chengdu, China; ^3^Department of Infection Control, West China Hospital, Sichuan University, Chengdu, China; ^4^Center for Pathogen Research, West China Hospital, Sichuan University, Chengdu, China

**Keywords:** carbapenemase, aminoglycosides, apramycin, streptomycin, neomycin, paromomycin, susceptibility, Enterobacteriaceae

## Abstract

We determined the *in vitro* susceptibility of four aminoglycosides, which are not of the 4,6-disubstituted deoxystreptamine (DOS) subclass against a collection of carbapenem-resistant Enterobacteriaceae (CRE). CRE clinical strains (*n* = 134) were collected from multiple hospitals in China and carried *bla*_NDM_ (*bla*_NDM−1_, *bla*_NDM−5_ or *bla*_NDM−7_; *n* = 66), *bla*_KPC−2_ (*n* = 62) or *bla*_IMP−4_ (*n* = 7; including one carrying *bla*_NDM−1_ and *bla*_IMP−4_). MICs of neomycin, paromomycin, streptomycin and apramycin as well as three 4,6-disubstituted DOS aminoglycosides (amikacin, gentamicin and tobramycin) were determined using the broth microdilution with breakpoints defined by the Clinical Laboratory Standards Institute (for amikacin, gentamicin and tobramycin), US Food and Drug Administration (streptomycin), the National Antimicrobial Resistance Monitoring System (apramycin) or la Société Française de Microbiologie (neomycin and paromomycin). Apramycin-resistant strains were subjected to whole genome sequencing using Illumina X10 platform. Among CRE strains, 65.7, 64.9, 79.1, and 95.5% were susceptible to neomycin (MIC_50_/MIC_90_, 8/256 μg/ml), paromomycin (4/>256 μg/ml), streptomycin (16/256 μg/ml) and apramycin (4/8 μg/ml), respectively, while only 55.2, 28.4, and 35.1% were susceptible to amikacin (32/>256 μg/ml), gentamicin (128/>256 μg/ml) and tobramycin (64/>256 μg/ml), respectively. Six CRE strains including five *Escherichia coli* of different sequence types and one *Klebsiella pneumoniae* were resistant to apramycin and the apramycin-resistant gene *aac(3)-IVa* was detected in all of these strains. In conclusion, neomycin, paromomycin, streptomycin and apramycin retain activity against most CRE strains. Although none of these non-4,6-disubstituted DOS aminoglycosides are suitable for intravenous use in human at present, these agents warrant further investigations to be used against CRE infections.

## Introduction

Carbapenem-resistant Enterobacteriaceae (CRE) have emerged as a major worldwide challenge for clinical treatment and public health (Temkin et al., 2014; Iovleva and Doi, 2017; Logan and Weinstein, 2017). The production of carbapenem-hydrolyzing β-lactamase enzymes (carbapenemases) is the major mechanism for carbapenem resistance in the Enterobacteriaceae. A variety of acquired carbapenemases of Class A (e.g., KPC and some GES enzymes), Class B (e.g., IMP, NDM, and VIM) and Class D (e.g., OXA-48 and its closely related enzymes) have been identified in CRE (Temkin et al., 2014; Iovleva and Doi, 2017; Logan and Weinstein, 2017). Globally, KPC, NDM, and OXA-48 are the most commonly encountered carbapenemases (Iovleva and Doi, 2017; Logan and Weinstein, 2017), while KPC-2, NDM-1, and NDM-5 are the most common ones in China including Sichuan province according to a multi-center survey (Zhang et al., 2017). *Klebsiella pneumoniae* is the most common species of CRE (Iovleva and Doi, 2017; Logan and Weinstein, 2017; Zhang et al., 2017).

The antimicrobial options against CRE are very limited (Van Duin et al., 2013). Colistin is the last resort antimicrobial agent commonly used to treat CRE infections but colistin resistance among CRE has also emerged worldwide (Poirel et al., 2017). Therefore, it is crucial to find antimicrobial agents as alternative choices. As very few new antimicrobial agents will most likely become available in the near future, repurposing currently available agents is another yet more realistic option. Aminoglycosides that are commonly used to treat clinical infections caused by bacteria other than *Mycobacterium* spp. include amikacin, gentamicin and tobramycin, all of which belong to the 4,6-disubstituted deoxystreptamine (DOS) subclass (Mingeot-Leclercq et al., 1999). These aminoglycosides retain activities against certain CRE strains (Livermore et al., 2011b) and their combination with other agents has been successful for treating some CRE infection cases (Hirsch and Tam, 2010; Falagas et al., 2014; Rafailidis and Falagas, 2014; Shields et al., 2016). Nonetheless, resistance to these 4,6-disubstituted DOS aminoglycosides among CRE is extensive (Livermore et al., 2011b; Almaghrabi et al., 2014; Smith and Kirby, 2016). In addition, the emergence of acquired 16S rRNA methylases including ArmA, RmtA to RmtH and NmpA, which confer high-level resistance to the 4,6-disubstituted DOS aminoglycosides, imposes another serious challenge for clinical management (Doi et al., 2016). Many CRE, in particular those carrying *bla*_NDM_ carbapenemase gene, have 16S rRNA methylase genes (Livermore et al., 2011b). Neomycin and paromomycin (also called neomycin E) belong to the 4,5-disubstituted DOS subclass (Mingeot-Leclercq et al., 1999), while apramycin is of the 4-monosubstituted DOS subclass and streptomycin is a non-DOS aminoglycoside as it contains an aminocyclitol other than DOS (Mingeot-Leclercq et al., 1999). Streptomycin is not affected by all 16S rRNA methylases, while the 4,5-disubstituted or 4-monosubstituted DOS aminoglycosides are only affected by NpmA (Doi et al., 2016), which is not common in the Enterobacteriaceae. Therefore, the non-4,6-disubstituted DOS aminoglycosides, which are usually not included into the panel for susceptibility tests or even are not approval for human use, may be potent against CRE. Here we present susceptibility results of four non-4,6-disubstituted DOS aminoglycosides (apramycin, streptomycin, neomycin and paromomycin) against 134 CRE clinical strains that were collected at multiple hospitals in China.

## Methods

### Bacterial strains

Non-duplicate CRE clinical strains (*n* = 134) from discrete patients were consecutively collected at 17 hospitals in 11 cities of Sichuan Province, western China since June 2016 to April 2017. Species identification was performed using the Vitek II system (bioMerieux, Durham, NC, USA). Acquired carbapenemase-encoding genes *bla*_GES_, *bla*_KPC_, *bla*_IMP_, *bla*_NDM_, *bla*_OXA−48_-like, and *bla*_VIM_ were screened for CRE strains using PCR as described previously (Poirel et al., 2000; Bradford et al., 2004; Mendes et al., 2007; Zong and Zhang, 2013). The specific allelic variants of the carbapenemase genes were obtained using PCR with additional primers able to amplify the whole encoding sequence (Zhang et al., 2012; Zong and Zhang, 2013) and Sanger sequencing.

### Antimicrobial susceptibility testing

MICs of aminoglycosides (amikacin, gentamicin, tobramycin, apramycin, neomycin, paromomycin, and streptomycin), ciprofloxacin, imipenem, meropenem, piperacillin-tazobactam and trimethoprim-sulfamethoxazole were determined using broth microdilution following the recommendations of the Clinical Laboratory Standards Institute (CLSI) (CLSI, 2017). Concentrations of these agents ranged from 0.5 to 256 μg/ml except for trimethoprim-sulfamethoxazole. *Escherichia coli* ATCC 25922 was used as the quality control and all tests were performed in triplicate. Breakpoints defined by CLST for amikacin, gentamicin and tobramycin (for amikacin, susceptible [S] ≤16 μg/ml, intermediate [I] 32 μg/ml, resistant [R], ≥64 μg/ml; for gentamicin and tobramycin, S ≤4 μg/ml, I 8 μg/ml, R ≥16 μg/ml), ciprofloxacin, imipenem, meropenem, piperacillin-tazobactam and trimethoprim-sulfamethoxazole (CLSI, 2017) was used, while no CLSI- or the European Committee on Antimicrobial Susceptibility Testing (EUCAST)-defined breakpoints for the other four agents are available. Breakpoints defined by US Food and Drug Administration (FDA) or the National Antimicrobial Resistance Monitoring System were used for streptomycin (S, ≤32 μg/ml; R, ≥64 μg/ml) and apramycin (S, ≤8 μg/ml; I, 16 or 32 μg/ml; R, ≥64 μg/ml) (Smith and Kirby, 2016), respectively. Those defined by Comite de L'Antibiogramme de la Société Française de Microbiologie (http://www.sfm-microbiologie.org/) were used for neomycin and paromomycin (S, ≤8 μg/ml; R, >16 μg/ml; for both agents).

### Genome sequencing and analysis

Genomic DNA of apramycin-resistant CRE strains were prepared using the QIAamp DNA Mini Kit (Qiagen, Hilden, Germany) and was subjected to whole genome sequencing with 200 × coverage using the HiSeq X10 Sequencer (Illumina, San Diego, CA). Reads were trimmed using Trimmomatic (Bolger et al., 2014) and were then assembled to contigs using the SPAdes program (Bankevich et al., 2012) with careful mode turned on. Antimicrobial resistance genes were identified from genome sequences using the ResFinder tool at the Center for Genomic Epidemiology (CGE, http://genomicepidemiology.org/) program. Sequence types were determined using the genomic sequence to query the multi-locus sequence typing (MLST) database using the MLST tool available at CGE.

### Nucleotide sequence accession numbers

Draft whole genome sequences of apramycin-resistant strains have been deposited in the DDBJ/EMBL/GenBank under accession numbers NGVA00000000, NGVB00000000, NMQY00000000, NTBE00000000, NTBF00000000, and NTBG00000000.

## Results

All 134 strains were confirmed as CRE as they were non-susceptible to imipenem and meropenem (MICs for both, 2 to >256 μg/ml). The 134 CRE strains included 1 *Citrobacter freundii*, 2 *Citrobacter koseri*, 13 *Enterobacter cloacae*, 25 *E. coli*, 1 *Klebsiella mobilis* (previously known as *Enterobacter aerogenes*), 3 *Klebsiella oxytoca*, 86 *Klebsiella pneumoniae* and 3 *Raoultella ornithinolytia* (Table 1). All of these CRE strains carried one or two carbapenemase-encoding genes, including *bla*_KPC−2_ in 62 strains, *bla*_NDM−1_ in 33, *bla*_NDM−5_ in 32, *bla*_NDM−7_ in 1 and *bla*_IMP−4_ in 7 with one *E. cloacae* carrying both *bla*_NDM−1_ and *bla*_IMP−4_ (Table 1).

**Table 1:** CRE strains and carbapenemase genes in the present study.

**Species**	***bla*_KPC−2_**	***bla*_NDM−1_**	***bla*_NDM−5_**	***bla*_NDM−7_**	***bla*_IMP−4_**	**Total**	**Distribution (no of hospitals)^a^**
*C. freundii*		1				1	1
*C. koseri*		2				2	1
*E. cloacae*		9^a^	3		2^b^	13	9
*E. coli*		3	21	1		25	8
*K. mobilis*		1				1	1
*K. pneumoniae*	58	16	8		4	86	15
*K. oxytoca*	2	1				3	3
*R. ornithinolytia*	2				1	3	2
Total	62	33^1^	32	1	7^a^	134	17

a *The number of hospitals from which the strains were recovered*.

b *One isolate carried both bla_NDM−1_ and bla_IMP−4_*.

Among the 134 CRE strains, only 28.4% (38/134) were susceptible to gentamicin and 35.1% (47/134) to tobramycin (Table 2), suggesting that they were not appropriate options for most CRE infections in our region. Slightly more than a half (55.2%, 74/134) of the CRE strains were susceptible to amikacin with the MIC_50_ and MIC_90_ being 32 μg/ml and >256 μg/ml, respectively (Table 2).

**Table 2:** *In vitro* antimicrobial susceptibilities of CRE strains.

	**MIC range (μg/ml)**	**MIC_50_ (μg/ml)**	**MIC_90_ (μg/ml)**	**Susceptible**	**Intermediate**	**Resistant**
**TOTAL (*****n*** = **134)**						
Amikacin	≤0.5->256	32	>256	74 (55.2%)	4 (3.0%)	56 (41.8%)
Gentamicin	≤0.5->256	128	>256	38 (28.4%)	8 (6.0%)	88 (65.7%)
Tobramycin	≤0.5->256	64	>256	47 (35.1%)	15 (11.2%)	72 (53.7%)
Apramycin	0.5->256	4	8	128 (95.5%)	–	6 (4.5%)
Neomycin	0.5->256	8	256	88 (65.7%)	–	46 (34.3%)
Paromomycin	0.5->256	4	>256	87 (64.9%)	–	47 (35.1%)
Streptomycin	0.5->256	16	256	106 (79.1%)	–	28 (20.9%)
Imipenem	2->256	64	256	0 (0.0%)	2 (1.4%)	132 (98.6%)
Meropenem	8->256	256	>256	0 (0.0%)	0 (0.0%)	134 (100%)
Piperacillin-tazobactam	8/4->256/4	>256/4	>256/4	1 (0.7%)	2 (1.5%)	131 (97.8%)
Ciprofloxacin	≤0.5->256	128	>256	29 (21.6%)	5 (3.7%)	100 (74.6%)
Trimethoprim-sulfamethoxazole	≤0.5/9.5- >128/2432	>128/2432	>128/2432	31 (23.1%)	–	103 (76.9%)
***K. pneumoniae*** **(*****n*** = **86)**						
Amikacin	1->256	>256	>256	38 (44.2%)	3 (3.5%)	45 (52.3%)
Gentamicin	≤0.5->256	256	>256	25 (29.1%)	1 (1.2%)	60 (69.8%)
Tobramycin	≤0.5->256	256	>256	28 (32.6%)	8 (9.3%)	50 (58.1%)
Apramycin	0.5->256	4	8	85 (98.8%)	0 (0.0%)	1 (1.2%)
Neomycin	0.5–256	4	256	55 (64.0%)	–	31 (36.0%)
Paromomycin	0.5->256	4	>256	54 (62.8%)	–	32 (37.2%)
Streptomycin	0.5->256	8	64	81 (94.2%)	–	5 (5.8%)
Imipenem	2->256	128	256	0 (0.0%)	1 (1.2%)	85 (98.8%)
Meropenem	8->256	256	>256	0 (0.0%)	0 (0.0%)	86 (100%)
Piperacillin-tazobactam	8/4->256/4	>256/4	>256/4	1 (1.2%)	2 (2.3%)	83 (96.5%)
Ciprofloxacin	≤0.5->256	128	>256	20 (23.3%)	3 (3.5%)	63 (73.3%)
Trimethoprim-sulfamethoxazole	≤0.5/9.5->128/2432	>128/2432	>128/2432	20 (23.3%)	–	66 (76.7%)
***E. coli*** **(*****n*** = **25)**						
Amikacin	2->256	16	>256	18 (72.0%)	0 (0.0%)	7 (28.0%)
Gentamicin	≤0.5->256	64	>256	5 (20.0%)	3 (12.0%)	17 (68.0%)
Tobramycin	≤0.5->256	32	>256	8 (32.0%)	3 (12.0%)	14 (56.0%)
Apramycin	1->256	8	>256	20 (80.0%)	0 (0.0%)	5 (20.0%)
Neomycin	1->256	8	256	15 (60.0%)	–	10 (40.0%)
Paromomycin	1->256	8	>256	14 (56.0%)	–	11 (44.0%)
Streptomycin	2->256	128	>256	12 (48.0%)	–	13 (52.0%)
Imipenem	8->256	32	128	0 (0.0%)	0 (0.0%)	25 (100%)
Meropenem	32->256	256	>256	0 (0.0%)	0 (0.0%)	25 (100%)
Piperacillin-tazobactam	>256/4	>256/4	>256/4	0 (0.0%)	0 (0.0%)	25 (100%)
Ciprofloxacin	0.5->256	>256	>256	1 (4.0%)	0 (0.0%)	24 (96.0%)
Trimethoprim-sulfamethoxazole	≤0.5/9.5- 128/2432	128/2432	128/2432	3 (12.0%)	–	22 (88.0%)
***E. cloacae*** **(*****n*** = **13)**						
Amikacin	1->256	8	>256	9 (69.2%)	1 (7.7%)	3 (23.1%)
Gentamicin	0.5->256	32	>256	2 (15.4%)	4 (30.8%)	7 (53.8%)
Tobramycin	1->256	16	>256	5 (38.5%)	4 (30.8%)	4 (30.8%)
Apramycin	1–4	4	8	13 (100%)	0 (0.0%)	0 (0.0%)
Neomycin	0.5->128	8	128	11 (84.6%)	–	2 (15.4%)
Paromomycin	0.5–256	4	256	11 (84.6%)	–	2 (15.4%)
Streptomycin	2->256	128	>256	4 (30.8%)	–	9 (69.2%)
Imipenem	4–128	32	128	0 (0.0%)	0 (0.0%)	13 (100%)
Meropenem	8–256	128	256	0 (0.0%)	0 (0.0%)	13 (100%)
Piperacillin-tazobactam	>256/4	>256/4	>256/4	0 (0.0%)	0 (0.0%)	13 (100%)
Ciprofloxacin	≤0.5->256	32	>256	3 (23.1%)	1 (7.7%)	9 (69.2%)
Trimethoprim-sulfamethoxazole	≤0.5/9.5->128/2432	>128/2432	>128/2432	3 (23.1%)	–	10 (76.9%)
***bla***_KPC−2_**-CARRYING STRAINS (*****n*** = **62)**						
Amikacin	≤0.5->256	>256	>256	18 (29.0%)	2 (3.2%)	42 (67.7%)
Gentamicin	≤0.5->256	>256	>256	9 (14.5%)	0 (0.0%)	53 (85.5%)
Tobramycin	≤0.5->256	256	>256	10 (16.1%)	5 (8.1%)	47 (75.8%)
Apramycin	0.5–8	4	8	62 (100%)	0 (0.0%)	0 (0.0%)
Neomycin	0.5–256	8	256	36 (58.1%)	–	26 (41.9%)
Paromomycin	0.5->256	8	>256	36 (58.1%)	–	26 (41.9%)
Streptomycin	0.5–64	8	32	61 (98.4%)	–	1 (1.6%)
Imipenem	8->256	128	>256	0 (0.0%)	0 (0.0%)	62 (100%)
Meropenem	8->256	>256	>256	0 (0.0%)	0 (0.0%)	62 (100%)
Piperacillin-tazobactam	256/4- >256/4	>256/4	>256/4	0 (0.0%)	0 (0.0%)	62 (100%)
Ciprofloxacin	≤0.5->256	128	>256	5 (8.1%)	2 (3.2%)	55 (88.7%)
Trimethoprim-sulfamethoxazole	≤0.5/9.5- >128/2432	>128/2432	>128/2432	11 (17.7%)	-	51 (82.3%)
***bla***_NDM_**-CARRYING STRAINS (*****n*** = **66)**						
Amikacin	1->256	8	>256	51 (77.3%)	2 (3.0%)	13 (19.7%)
Gentamicin	≤0.5->256	16	>256	26 (39.4%)	8 (12.1%)	32 (48.5%)
Tobramycin	≤0.5->256	8	>256	33 (50.0%)	9 (13.6%)	24 (36.4%)
Apramycin	1->256	4	8	60 (90.9%)	0 (0.0%)	6 (9.1%)
Neomycin	0.5->256	8	256	46 (69.7%)	–	20 (30.3%)
Paromomycin	0.5->256	4	>256	45 (68.2%)	–	21 (31.8%)
Streptomycin	0.5->256	16	>256	40 (60.6%)	–	26 (39.4%)
Imipenem	4->256	64	128	0 (0.0%)	0 (0.0%)	66 (100%)
Meropenem	8->256	128	>256	0 (0.0%)	0 (0.0%)	66 (100%)
Piperacillin-tazobactam	256/4- >256/4	>256/4	>256/4	0 (0.0%)	0 (0.0%)	66 (100%)
Ciprofloxacin	0.5->256	>256	>256	7 (10.6%)	2 (3.0%)	57 (86.4%)
Trimethoprim-sulfamethoxazole	≤0.5/9.5- >128/2432	>128/2432	>128/2432	17 (25.8%)	–	49 (74.2%)

Slightly less than 2/3 of the CRE strains were susceptible to neomycin (65.9%, 88/134) and paromomycin (64.9%, 87/134), respectively (Table 2). Most CRE strains (106/134, 79.1%) were susceptible to streptomycin (MIC, ≤32 μg/ml) with its MIC_50_ and MIC_90_ of being 16 μg/ml and >128 μg/ml, respectively.

Among aminoglycosides tested, apramycin appears to be the most promising as its MICs against almost all (95.5%, 128/134) of the CRE strains were 8 or less (MIC_50_ and MIC_90_ was 4 and 8 μg/ml, respectively). Nonetheless, six CRE strains including five *E. coli* and one *K. pneumoniae* were resistant to apramycin, all of which were high-level resistance (MIC of apramycin, >256 μg/ml). Draft whole genomic sequences of these strains were obtained. A total of 4,453,184–7,526,752 clean reads and 1.34–2.26 Gb clean bases were generated for the six strains, which were then assembled to 106–229 contigs (79–187 were ≥1,000 bp in length) with a 50.25–50.57% GC content for *E. coli* and 57.03% for *K. pneumoniae*, respectively (Table 3). The five apramycin-resistant *E. coli* belonged to five sequence types (ST101, ST167, ST206, ST6388, and ST6823), suggesting that the apramycin-resistant strains were not clonal. The apramycin-resistant *K. pneumoniae* belonged to ST340. All of apramycin-resistant *E. coli* and *K. pneumoniae* had *aac(3)-IVa*, which encodes an aminoglycoside-modifying enzyme conferring resistant to apramycin (Shaw et al., 1993).

**Table 3:** Genomic characteristics of the six apramycin-resistant CRE strains.

**Isolate**	**Species**	**ST**	**Clean reads**	**Clean bases (Gb)**	**Contigs**	**% GC content**	**GenBank accession no**
WCHEC66	*E. coli*	ST6823	4,493,365	1.35	106	50.47	NGVB00000000
WCHEC68	*E. coli*	ST101	4,453,184	1.34	229	50.25	NGVA00000000
SCEC76	*E. coli*	ST167	7,526,752	2.26	192	50.52	NTBG00000000
SCEC88	*E. coli*	ST6388	6,197,204	1.86	222	50.57	NTBF00000000
WCHEC-LL123	*E. coli*	ST206	5,751,013	1.73	193	50.40	NMQY00000000
WCHKP118	*K. pneumoniae*	ST340	5,620,454	1.69	115	57.03	NTBE00000000

## Discussion

Neomycin is not given via injection due to its nephrotoxicity, while paromomycin is on the List of Essential Medicines of World Health Organization (World Health Organization, 2015) and is used to a number of parasite infections such as amebiasis and giardiasis by oral or intramuscular injection. Both neomycin and paromomycin are poorly absorbed when taken orally. Nonetheless, oral administration of neomycin has been used for inhibiting the overgrowth of gut microflora (Clark, 1977) and for decolonizing the intestinal carriage of extended-spectrum β-lactamase(ESBL)-producing Enterobacteriaceae (Huttner et al., 2013). Paromomycin has also been applied for decolonizing ESBL-producing Enterobacteriaceae (Buehlmann et al., 2011; Rieg et al., 2015). As both agents are not given by intravenous injection, they are unlikely to be used for treating systematic infections caused by CRE. However, our results suggest that neomycin and paromomycin deserve further investigations to be used enterally and to be modified to reduce their nephrotoxicity.

The fact that close to 80% of CRE strains were susceptible to streptomycin suggests that streptomycin may have a potential role in treating CRE infections. However, streptomycin is only available for intramuscular injection and therefore may not be appropriate for treating patients with critical illness. Nonetheless, the efficacy of streptomycin, probably in combination with other agents such as β-lactams, in treating CRE infections warrants to be explored.

The vast majority of CRE strains were susceptible to apramycin, suggesting the excellent *in vitro* activity against CRE. A previous study has found that 70.8% of 71 CRE clinical strains collected in USA were susceptible to apramycin (Smith and Kirby, 2016), while in another study 80 of 82 (97.6%) CRE clinical isolates, most of which were collected in UK, were susceptible to apramycin (Livermore et al., 2011a). These findings suggest that the excellent activity of apramycin against CRE is not geographically restricted. Unfortunately, apramycin is a veterinary agent and has not been approved for clinical use, which is likely due to its narrow therapeutic index (Livermore et al., 2011a). The use of aminoglycosides has been limited by its toxicity, particularly nephrotoxicity and ototoxicity. However, apramycin has low ototoxicity (Matt et al., 2012) and fewer nephrotoxic side effects (Kostrub et al., 2009; Kang et al., 2017) as it appears to have higher affinity to bacterial over mitochrondrial ribosomes (Perzynski et al., 1979; Kang et al., 2017). In clinical settings, aminoglycosides are commonly used in combination with other antimicrobial agents, particularly β-lactams and sometimes fluoroquinolones. There are no data about the combination of these non-4,6-disubstituted DOS aminoglycosides with other agents against CRE at present. Nonetheless, in animal models the combination of apramycin and a fluoroquinolone (enrofloxacin) shows synergic effect to increase the efficacy against Gram-negative bacilli such as *E. coli* and *Salmonella* and is also able to decrease the emergence of mutational resistance to fluoroquinolones (Randall et al., 2016). Apramycin therefore warrants further investigations as a repurposed agent against CRE. The unusual structure of apramycin also provides a scaffold for further modification to generate new potent and safe compounds for treating CRE infections (Livermore et al., 2011a).

Although our results suggest that the non-4,6-disubstituted DOS aminoglycosides had good *in vitro* activities against CRE, the results should be interpreted with cautions. First, unlike 4,6-disubstituted DOS aminoglycosides, the susceptibility interpretation (breakpoints of MICs) of the four non-4,6-disubstituted DOS aminoglycosides against Enterobacteriaceae has not been well established and validated. In particular, apramycin is a veterinary agent and its susceptibility/resistance breakpoints may not be suitable for human medicine. Second, there are toxicity concerns, particularly nephrotoxicity, related to the four non-4,6-disubstituted DOS aminoglycosides and none of the four agents are suitable for intravenous use in human at present. Additional studies must be carried out to address any safety risks. Third, development of resistance to the four aminoglycosides also poses a huge challenge for their potential use against CRE. For instance, high level resistance to streptomycin can be rapidly developed (Sinha, 1986).

In conclusion, most (64.9–95.5%) CRE strains in this collection were susceptible to neomycin, paromomycin, streptomycin or apramycin, while less strains (28.4–55.2%) were susceptible to the mainstream clinically-available aminoglycosides amikacin, gentamicin and tobramycin. In particular, almost all CRE strains were susceptible to apramycin, suggesting that apramycin may be an excellent candidate for modification to generate new potent and safe aminoglycosides. Although none of these non-4,6-disubstituted DOS aminoglycosides are suitable for intravenous use in human at present, they warrant further investigation for treating CRE infections.

## Author contributions

ZZ designed the experiments, analyzed the data and wrote the manuscript. YH and LL performed the experiments and analyzed the data. XZ and YF contributed to analyzing the data and co-wrote the manuscript.

### Conflict of interest statement

The authors declare that the research was conducted in the absence of any commercial or financial relationships that could be construed as a potential conflict of interest.
